# Mean nutrient adequacy ratio and associated factors of complementary foods among children aged 6–23 months in Northeast Ethiopia

**DOI:** 10.3389/fped.2025.1446431

**Published:** 2025-03-07

**Authors:** Abdulkerim Kassaw, Tefera Chane Mekonnen, Erkihun Tadesse Amsalu, Eyob Tilahun ABeje, Chala Daba, Yawkal Tsega, Abel Endawkie

**Affiliations:** ^1^ Masters of Public Health Nutrition, School of Public Health, College of Medicine and Health Sciences, Wollo University, Dessie, Ethiopia; ^2^ Department of Public Health Nutrition, School of Public Health, College of Medicine and Health Sciences, Wollo University, Dessie, Ethiopia; ^3^ Department of Epidemiology and Biostatistics, School of Public Health, College of Medicine and Health Sciences, Wollo University, Dessie, Ethiopia; ^4^ Department of Environmental Health, College of Medicine and Health Sciences, Wollo University, Dessie, Ethiopia; ^5^ Department of Health System and Management, School of Public Health, College of Medicine and Health Sciences, Wollo University, Dessie, Ethiopia

**Keywords:** mean nutrient adequacy ratio, children, northeast, Ethiopia, complementary foods, associated factors

## Abstract

**Background:**

Inadequate intake of macro- and micronutrients is a significant public health challenge in Ethiopia. Ethiopia carries a substantial burden of both macro- and micronutrient deficiencies, with far-reaching consequences. However, there is limited evidence on the determinants and mean nutrient adequacy of complementary foods among children aged 6–23 months. Therefore, this study aims to determine the mean nutrient adequacy ratio and associated factors of complementary feeding among children aged 6–23 months in Northeast Ethiopia.

**Methods:**

A study was conducted in Northeast Ethiopia between 1 March and 30 April 2023, among 255 children aged 6–23 months. The study employed a systematic sampling technique to select 255 children. The data were collected using a 24-h dietary recall method. Linear regression analysis was conducted to identify factors associated with the nutrient adequacy of complementary foods. In the multivariable analysis, variables with *p*-values <0.05 were considered statistically significant.

**Results:**

The study found that the mean nutrient adequacy ratio of complementary foods among children aged 6–23 months in Northeast Ethiopia was 63% (95% CI 60.8–65.14). The adequacy of nutrients in complementary foods varied: energy (90%), fat (93%), carbohydrate (70%), protein (88%), calcium (57%), zinc (52%), vitamin B1 (50%), vitamin A (52%), and vitamin C (60%). The age of the child, mother's education, wealth index, feeding frequency, dietary diversity, minimum acceptable diet, source of information during antenatal care, postnatal care, food insecurity, and the number of children aged under 5 were associated with mean nutrient adequacy of complementary feeding.

**Conclusions:**

The study concludes that the mean nutrient adequacy ratio of complementary foods among children aged 6–23 months in Northeast Ethiopia is alarmingly low, indicating a significant gap in meeting the nutritional recommendations set by the World Health Organization. Various factors, including the child's age, maternal education, household wealth index, food insecurity, and dietary diversity, were identified as critical determinants of nutrient adequacy. These findings underscore the urgent need for comprehensive interventions at multiple levels, including education, food security, and health services, to enhance the consumption of adequate and diverse complementary foods. By addressing these factors, stakeholders can improve the nutritional status of young children, ultimately contributing to better health outcomes and development in the region.

## Introduction

Complementary foods are essential for child growth, development, and survival in young children aged 6–23 months (1). Complementary foods should contain high-biological value protein, vitamins, and minerals (2). Nutritional adequacy is the comparison between the requirement and intake of nutrients of a certain individual and the mean nutrient adequacy ratio (MNAR) used to evaluate the general adequacy of the diet from food and beverage intake (3). Globally, micronutrient inadequacy is a major public health problem, with more than 340 million children experiencing inadequacy of essential micronutrients and at least 1 in 2 children with hidden hunger (4). In Ethiopia, protein-energy malnutrition and micronutrient deficiency, such as iron, vitamin A, zinc, and iodine deficiencies, are major nutritional problems (5, 6). Inadequate nutrient intake in children aged below 2 years results in growth faltering as long-term effects are poor cognitive development, reproductive dysfunction, poor economic productivity, and nutrition-related chronic diseases (7). However, MNAR is associated with various factors, such as adequacy of nutrient contents and dietary diversity (8). Every food group contains a particular nutrient and each nutrient has a specific function in the human body; therefore, consumption of various foods in the right proportion from different food groups is vital to the well-being of individuals (8).

In Ethiopia, various studies have shown that nutrient adequacy among children aged 6–23 months is influenced by factors such as family size, food group, number of children, antenatal (ANC) and postnatal care (PNC), food security, feeding frequency, dietary diversity, nutrient content, childhood illness, infant age, adequate care, parental education and occupation, and resident-related characteristics (9–13). However, the mean nutrient adequacy ratio of complementary foods and its associated factors have not been well studied among children aged 6–23 months, particularly in northeast Ethiopia. Therefore, this study aimed to assess the mean nutrient adequacy ratio and factors influencing complementary foods among children aged 6–23 months in this region.

## Methods

### Study setting and period

This study was conducted in Kombolcha, Northeast Ethiopia, between 1 March and 30 April 2023. According to the 2022 Kombolcha administration health office report, the town has a total population of approximately 156,140 people, distributed across 12 administrative kebeles, with 12 health posts, four health centers, and seven private medium clinics that provide health services, including maternal and child healthcare and one general hospital. The number of infants and young children aged 6–23 months is estimated to be 6,761 of the total population.

### Study design and population

The study was conducted using a community-based cross-sectional study design. The study included all children aged 6–23 months with mothers who had lived in the selected kebeles for at least 6 months.

### Sample size determination and sampling methods

**Figure 1:**
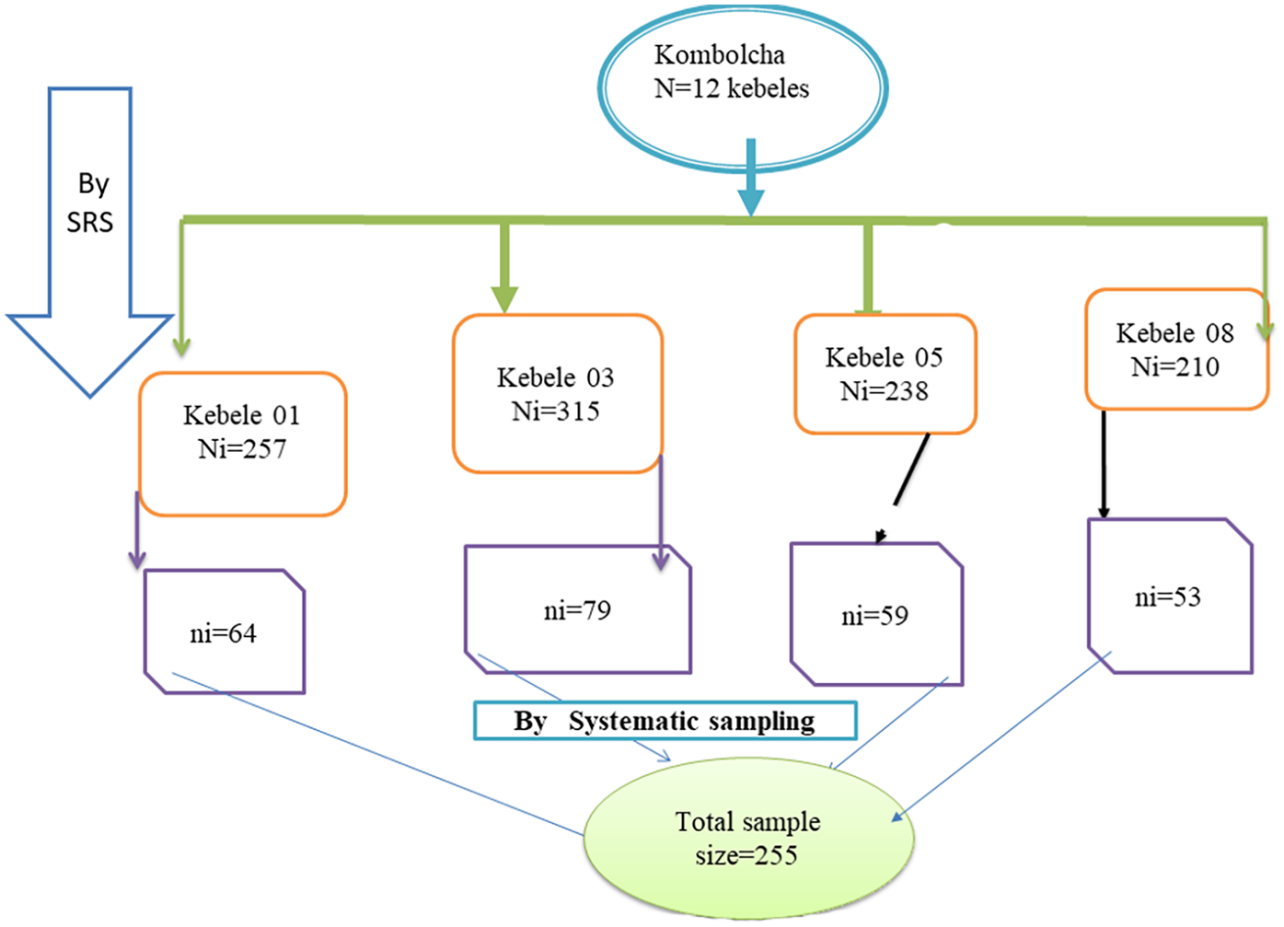
Schematic presentation of sampling procedure in Kombolcha, Northeast Ethiopia, 2023.

The sample size was calculated using Stata version 14 software, with a single population proportion formula with the following assumptions: Alpha = 0.05, Power = 0.8, Delta = 0.1850, mean adequacy ratio (MAR) H0 = 0.67, Ha = 0.7070, and (MAR) SD = 0.2001 (14). After accounting for non-response, the final sample size was 255. Initially, there were 12 kebeles, out of which four kebeles were selected using a simple random sampling (SRS) lottery method. A sampling frame containing 1,016 lists of households with children aged 6–23 months was obtained and listed using the Kebele health post registration log book. All listed households were coded with a numeric code. The sample size was allocated to the population size proportionately, and the sampling interval (*K* = 4) was calculated. Finally, 255 participants were selected using a systematic sampling method after randomly identifying the first household and proceeding to the second participant based on the interval. The number of infants drawn from each kebele was calculated by proportion using the formula ni = Ni*n/*N*, where *N* is the total number of infants in 12 districts, Ni is the number of infants in each district, *n* is the total sample size to be selected in kebeles, and ni is the number of infants drawn from each kebele (Figure 1).

### Study variables

The mean nutrient adequacy ratio of complementary food among children aged 6–23 months was a dependent variable along with sociodemographic factors, including child’s age, child’s sex, household size, marital status, parental education and occupation, mother’s age and wealth index. Independent variables included the following: food security factors, such as food access, food group and food availability; maternal and child health factors, such as breastfeeding status, maternal care, feeding frequency, nutrients intakes of macro and micronutrient intake; and medical and healthcare service-related factors, such as child disease, child care, ANC and PNC visits.

### Variable measurement

#### The mean adequacy ratio

The MAR was computed as an overall measure of dietary intake adequacy (15). A mean adequacy ratio value to reflect indexes of dietary quality (15).

#### Household food security

The Household Food Insecurity and Access Scale (HFIAS) is a continuous measure assessing the degree of food insecurity based on the frequency of nine food insecurity-related events in the preceding 4 weeks (30 days). Household scores are in the range of 0–27, with higher scores indicating greater food insecurity. Based on this scale, food insecurity is classified into four categories: food secure (0–1), mild (2–8), moderate (9–14), and severe (15–27) (2, 16).

#### Minimum dietary diversity

The proportion of children aged 6–23 months who received foods from five or more of the eight food groups was calculated. The eight food groups used for tabulation of this indicator were breast milk grains, legumes, dairy products, flesh foods, eggs, vitamin A-rich fruits and vegetables, and other fruits and vegetables (2).

#### Minimum meal frequency

The proportion of breastfed children aged 6–23 months who received solid, semi-solid, or soft foods the minimum number of times or more was calculated. The adequate minimum meal frequency for children aged 6–9 months, 9–11, and 11–23 months is 2–3 meals, more than 3 meals, and 3–4 meals per day, respectively. For a non-breastfed child, it should be at least four times in the last 24 h (2).

#### Minimum acceptable diet

The proportion of breastfed and non-breastfed children aged 6–23 months who had at least the minimum dietary diversity and minimum meal frequency during the previous day was calculated (2).

#### Wealth index

The wealth index was computed using composite asset indicators for urban residents. A composite wealth index was constructed using principal components analysis (PCA) based on asset indicators, including television, refrigerator, mobile telephone, availability of electric power, fixed phone, bicycle, car, and cart. The final wealth index was classified into five categories: Lowest, Second, Middle, Fourth, and Highest (17).

### Data collection techniques and tools

The data collection for this study was conducted by interviewing mothers or primary caregivers of children aged 6–23 months in their own homes. The data collection instrument was a structured questionnaire prepared in English. To collect dietary intake data, 24-h dietary recall was conducted with the caregivers of the children using the multiple pass technique. This technique includes a quick list of consumed food, a detailed description of the listed food, and a review of the recall if the respondent may have forgotten the recall. In some situations, siblings and fathers of the child assisted the mother in recalling the last 24-h food intake of the child. All days of the week were equally represented in the final sample. All the recall days were arranged on non-special occasions like holidays, feast days, death occasions in the household, or fasting time. On the interview day, the participants were requested to report all foods that they served their child on the previous day, using a multiple-pass method.

Data were collected by six diploma nurses who had experience in collecting data in urban settings, were native speakers of the local languages, and were intensively trained for this study. Five days before the interview, the principal investigator visited 20 households to collect data on common child food, ingredients, and cooking methods and took photos of equipment commonly used to serve food for children. The food list was used to probe for food intake and describe the ingredients of listed food during data collection. It was read to the participant after completing dietary recall to help her recall any food that she forgot to list (Supplementary File I). The equipment from the pictures was purchased from the local.

We use salted replicas of local common foods for infants and young children to estimate portion size. Portions were mostly estimated by direct weighing of salted replicas of actual foods prepared locally. The salted replicas consisted of injera (fermented flat, pancake), shiro (a legume-based spicy stew), and bread. Otherwise, graduated food models and common household measures were used. In most cases, siblings living market. The equipment included a spoon, cup, ladle, bowl, and feeding bottle.

To estimate the portion size of the actual foods, a dietary scale was used to weigh them. The locally available labeled cup, bowl, spoon, ladle, bottle, and food weighing scale were calibrated to estimate the portion size (Supplementary File II). The completeness of the information gathered was checked, and salted replicas of common local food for infants and young children were also used. Each participant was asked to put a quantity of food that was equivalent to the eaten food on the food weighing scale. If the actual food was not available in the house, they were asked to borrow from neighbors. Otherwise, they were asked to estimate the portion of food that their child ate using equipment handled by the data collector. Most of the dietary intake was weighed in the household of the respondent. For purchased food, the respondent was asked about the monetary value and labeling/brand of the food (Supplementary File III).

### Data quality control

Interviewers were trained before the actual data collection. They were frequently supervised and the questionnaires were pretested on 10% of the participants in the selected areas. The questionnaire was then rechecked for its precision and consistency, and necessary modifications were incorporated before commencing the actual data collection. Similarly, interviews were conducted in local languages. The English version of the questionnaire was translated first to Amharic and back to English by the language experts to maintain consistency. The selected data collectors received 2 days of training before data collection and two BSc nurses were recruited as supervisors.

### Data processing and analysis

#### Estimation of nutrient adequacy of complementary foods

The compiled food composition tables were entered into the software package, food processor (version 8.1) to create a dietary database. The dietary data were then entered into this software to calculate the nutrient composition of the food. An ESHA food processor was used to calculate the nutrient values of the food, except for Ethiopia's local foods, which were calculated based on their values from Ethiopia's food composition table. Missing values were completed using the Kenya nutrient database and the West African food composition table (18, 19). Two canned mango juices (RAN and YAMI) were not labeled for their nutrient intake. For this reason, we borrowed canned mango juice from USDA Sir 26 to calculate their nutrient intake. The data were analyzed and converted to the amount of nutrient and energy intake per individual per day. The NAR of macro- and micronutrient intakes from complementary foods were calculated for children aged 6–23 months and compared with the World Health Organization’s (WHO) recommended dietary allowance (RDA) (20). Moderate (10%) and low bioavailability (15%) were assumed to assess the adequacy of zinc and iron.

Data entry was made using EPi-Data software and exported to SPSS 23 for analysis. Frequency, percentage, mean, and standard deviation were used to summarize the data. Food composition tables were analyzed using a food processor (version 8.1).

The MAR was used as an overall measure of dietary adequacy. To compute MAR, the nutrient adequacy ratio (NAR) has to be calculated, which is deﬁned as the ratio of an individual's nutrient intake to the recommended nutrient intake (RNI) of the assessed nutrient, appropriate for the age and sex of the individual

(1) $$\mathrm{NAR}=\frac{\mathrm{Actual}\;\mathrm{nutrient}\;\mathrm{intake}\;(\mathrm{per}\;\mathrm{day})}{\mathrm{Recommended}\;\mathrm{nutrient}\;\mathrm{intake}}\times 100$$

The value for MAR is calculated as the total of all the NARs divided by the total expressed as a percentage. MAR is calculated in a range of 0%–100% (4)

(2) $$\mathrm{MAR}=\frac{\sum \mathrm{NARs}}{\mathrm{Total}\;\mathrm{number}\;\mathrm{of}\;\mathrm{nutrients}}\times 100$$

For computing MAR, each of the NARs was truncated as 1 so that a high intake of one nutrient could not compensate for the low intake of another nutrient, as originally conceived (15).

Linear regression analysis was used to determine an association between mean nutrient adequacy ratio (MAR) and its associated factors of complementary food among children aged 6–23 months in Northeast Ethiopia. Simple and multivariable linear regression analyses were used to model MAR and its associated factors. All explanatory variables that demonstrated a *p*-value <0.25 (21) in the simple analysis were considered candidates for the multivariable models. The outputs of the analyses are presented via crude and coefficient of determination (*β*). In final multivariable linear regression models and statistically significant at *p*-value <0.05, the extent of multicollinearity was measured using the variance inflation factor (VIF) and found to be within the variable range (the VIF for each variable was less than 10).

### Ethical consideration

Ethical approval was obtained from the Institutional Review Board of the Wollo University College of Medicine and Health Science, School of Public Health. A letter of permission was obtained from the Kombolcha Administrative Health Office. Each study participant provided written informed consent to confirm their willingness to participate in the research after being informed of the study's goal and purpose. Written informed consent was presumably obtained from legally authorized representatives, such as parents (mothers) and/or legal guardians (caregivers). In addition, participants were made aware that their participation in the study was entirely voluntary and that they might leave at any moment if the questionnaire did not feel comfortable. The participant's name was not entered into the questionnaire, and the data were password-protected and stored on a computer to maintain privacy and secrecy. During the data collection period, the study participants received critical advice regarding diet from the data collectors, and all methods and data were performed based on Helsinki principles and Ethiopian research guidelines.

## Results

### Sociodemographic characteristics of study respondents

A total of 255 mothers with children aged 6–23 months were interviewed, with a response rate of 100%. Of the children in the study, 56% were male. The mean (±SD) age of the children was 13.2 ± 4.7 months, and almost half (*n*=142, 55.7%) of them were in the age category of 12–23 months. In addition, two-thirds of the mothers (*n* = 143, 56.1%) were aged 25–34 years. Regarding maternal literacy, 196 (76.9%) mothers were formally educated (Table 1).

**Table 1:** Sociodemographic characteristics of respondents in Kombolcha, Northeast, Ethiopia, 2023.

Variables	Frequency (*n* = 255)	Percent
Sex of the child		
Female	112	43.9
Male	143	56.1
Childs age		
6–8 months	44	17.3
9–11 months	69	27
12–23 months	142	55.7
Mother's age		
15–24 years	49	19.2
25–34 years	143	56.1
35–49 years	63	24.7
Mothers marital status		
Married	241	94.5
Divorced	14	5.5
Mother's education status		
Informal education	59	23.1
Formal education	196	76.9
Father's educational status		
Informal education	27	10.6
Formal education	228	89.4
Mother's occupational status		
Government employed	16	6.2
Private/non-government employee	78	30.6
Merchant	24	9.4
Daily labor	14	5.5
Student	3	1.2
Unemployed	12	4.7
Housewife	102	40.0
House servant/worker	6	2.4
Father's occupational status		
Government employee	56	22.0
Private/non-government employee	78	30.6
Merchant	62	24.3
Daily labor	33	12.9
Student	2	.8
Unemployed	24	9.4
Family size		
<5 members	183	71.8
>5 members	72	28.2
Number of under-five children		
One	192	75.3
Two or more	63	24.7
Maternal relation with children		
Caregiver	17	6.7
Mother	238	93.3
Household wealth index		
Poorest	7	2.7
Poor	87	34.1
Middle	101	39.6
Rich	50	19.7
Richest	10	3.9

### Feeding practices for children aged 6–23 months and food security

**Figure 2:**
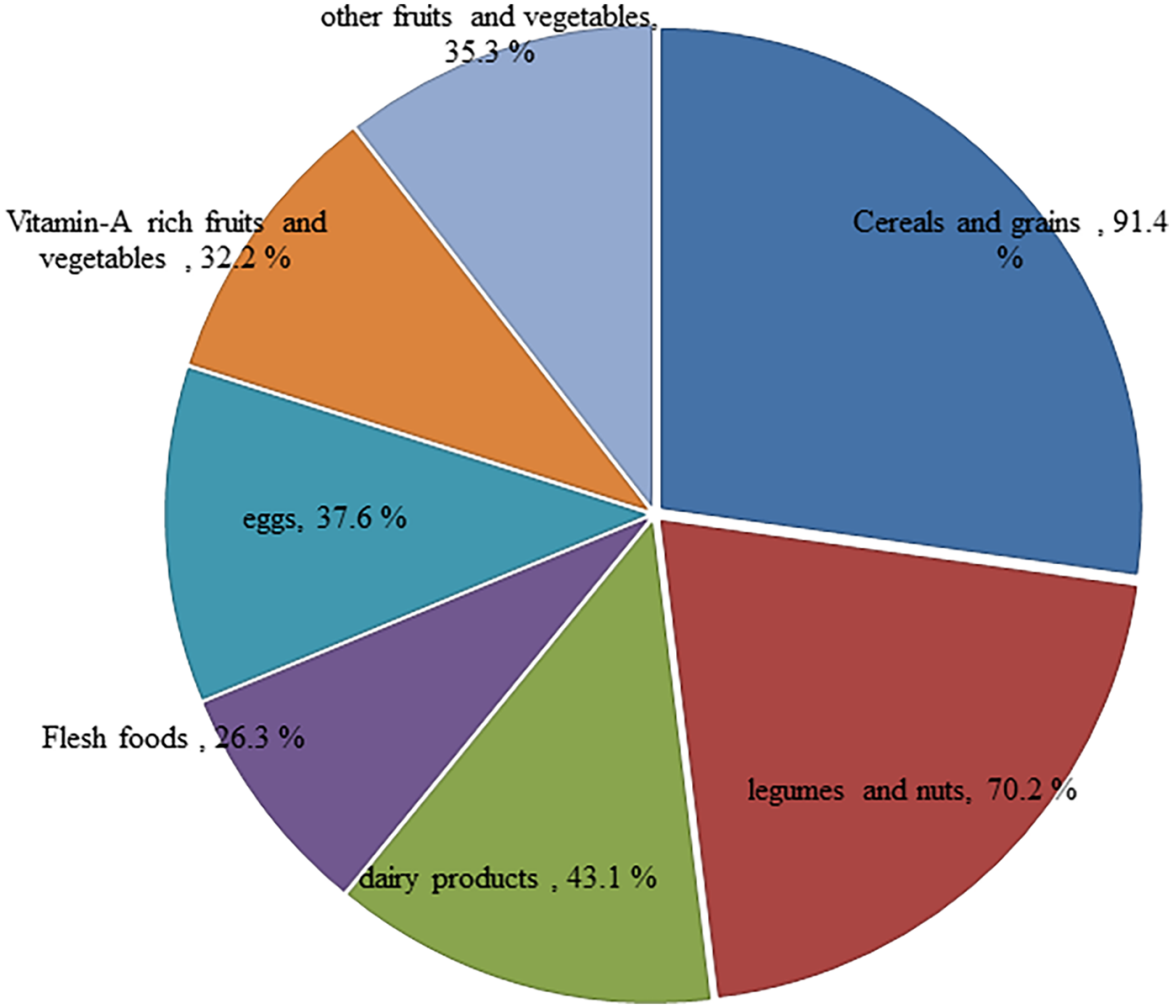
Types of food groups consumed among children aged 6–23 months in Northeast Ethiopia, 2023.

Among children aged 6–23 months in Northeast Ethiopia in 2023, 233 (91.4%) were eating cereals and grains, 179 (70.2%) legumes and nuts, 110 (43.1%) dairy products (milk, yogurt), 67 (26.3%) flesh foods (meat, fish, poultry), 96 (37.6%) eggs, 82 (32.2%) vitamin-A rich fruits and vegetables, and 90 (35.3%) other fruits and vegetables (Figure 2).

The majority of children (*n* = 208, 81.6%) were breastfed. Both early and late initiation of additional food were practiced extensively in the study area, but most (*n* = 212, 83.1%) of the mothers started to give complementary food to their children at just 6 months. Approximately 139 (54.5%) children met the minimum meal frequency requirement. The present study found that a minimum acceptable diet was consumed by 48 (18.8%) participants and 150 (58.8%) of them consumed less than four food groups per day in their recommended dietary diversity. Market purchasing was reported as the major source of food in 240 (94.1%) households. Furthermore, a food security assessment based on HFIAS revealed that 102 (40%) of the households were food secure (Table 2).

**Table 2:** Infant and young children feeding practices, food security, and food groups among children aged 6–23 months in Northeast Ethiopia, 2023.

Variables	Frequency (*n* = 255)	Percent
Currently breastfeeding		
Yes	208	81.6
No	47	18.4
Time of starting complementary food		
Before 6 months	20	7.8
At 6 months	212	83.1
After 6 months	23	9.1
Minimum meal frequency		
No	116	45.5
Yes	139	54.5
Minimum dietary diversity		
<Four food groups per day	150	58.8
≥Four food groups per day	105	41.2
Minimum acceptable diet		
No	207	81.2
Yes	48	18.8
Food security status		
Food secure	102	40.0
Mild insecurity	55	21.6
Moderate insecurity	57	22.4
Severe insecurity	41	16.0
Household food source		
Own/domestic production	15	5.9
Market purchasing	240	94.1

### Medical and healthcare information-related factors

Approximately 3%, 83%, 9%, and 6% of the mothers received dietary diversity-related information or education from health posts, health centers, hospitals, and private health organizations, respectively, in the month preceding the study. The majority received information about the importance of dietary diversity during ANC visits (*n* = 194, 76%) and PNC checkups (*n* = 204, 80%). In addition, the majority (*n* = 185, 72.5%) of mothers do not prepare any particular food for their children during sickness or recovery from disease (Table 3).

**Table 3:** Medical and healthcare information related factors among children aged 6–23 months in Kombolcha, Northeast Ethiopia, 2023.

Variables	Frequency (*n* = 255)	Percent
Received information about the importance of dietary diversity during the ANC visit		
No	61	23.9
Yes	194	76.1
Frequency of antenatal care visit		
1–3 Visited	34	13.3
>4 Visited	221	86.7
Source of information about dietary diversity		
health post	7	2.7
Health center	210	82.4
Hospital	22	8.6
Privet health organization	16	6.3
Received information about the importance of dietary diversity during the PNC visit		
No	51	20.0
Yes	204	80.0
Particular food during sickness or recovery		
No	185	72.5
Yes	70	27.5

### Adequate nutrients intake

The mean intake of macro- and micronutrients such as energy, fat, protein, carbohydrate, calcium, iron, zinc, vitamin A, vitamin C, vitamin B1, vitamin B2, niacin, vitamin B6, vitamin B12, and folate was significantly lower than the reference value for all age categories (Supplementary File III**).**

For all children aged 6–23 months, adequate nutrients in the complementary foods were energy (90%), fat (93%), carbohydrate (70%), protein (88%), calcium (57%), zinc (52%), vitamin B1 (50%), vitamin B2 (51%), vitamin B3 (52%), vitamin B6 (54%), vitamin B12 (54%), iron (54%), vitamin A (52%), vitamin C (60%), and folate (64%).

**Figure 3:**
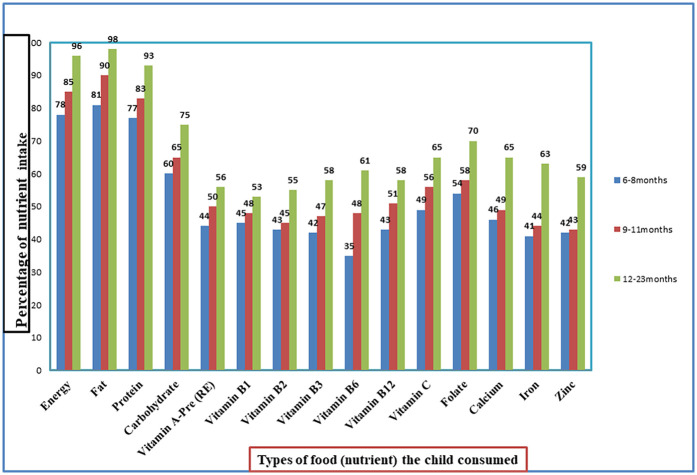
Nutrients intake among children aged 6–23 months in Northeast Ethiopia, 2023.

The nutrient adequacy of complementary foods for children aged 6–8 months was energy (78%), fat (81%), protein (77%), carbohydrate (60%), vitamin A (44%), vitamin B1 (45%), vitamin B2 (43%), vitamin B3 (42%), vitamin B6 (35%), vitamin B12 (43%), vitamin C (49%), folate (54%), calcium (46%), iron (41%), and zinc (42%). The nutrient adequacy of complementary foods for children aged 9–11 months was energy (85%), fat (90%), protein (83%), carbohydrate (65%), vitamin A (50%), vitamin B1 (48%), vitamin B2 (45%), vitamin B3 (47%), vitamin B6 (48%), vitamin B12 (51%), vitamin C (56%), folate (58%), calcium (49%), iron (44%), and zinc (43%). Finally, the mean nutrient adequacy ratio for children aged 6–23 months was 63% (Figure 3).

### Predictors of mean nutrient adequacy ratio (MNAR)

In this study, several factors were evaluated for their association with the mean nutrient adequacy ratio (MNAR) using the crude (bivariable) analysis. For example, the results revealed that boys had a coefficient of 16.99 (95% CI 13.38–20.61), indicating a significant positive association with MNAR. In addition, each month of age was linked to an increase in MAR of 1.33 (95% CI 0.92–1.74). Furthermore, the children of mothers who had a formal education demonstrated a significantly higher MAR, with a coefficient of 19.30 (95% CI 15.00–23.61). Conversely, the analysis showed a negative association with the number of children aged under 5 years, reflected in a coefficient of −27.75 (95% CI −31.06–−24.42). In this study, we considered age, socioeconomic status, and sex of the study participants as the natural confounders that may affect the relationship between independent and dependent variables.

To account for these confounders, a multivariable linear regression model was used. The extent of multicollinearity was measured using a variance inflation factor (VIF) and was found to be within the variable range (the VIF for each variable was less than 10). In the multivariable linear regression models, variables such as feeding frequency (*β*: 3.49; 95% CI 2.12–4.85), minimum dietary diversity (*β*: 9.74; 95% CI 7.66–11.82), minimum acceptable diet (*β*: 6.16; 95% CI 4.12–8.20), ANC follow-up (*β*: 3.26; 95% CI 1.23–5.29), and PNC follow-up (*β*: 2.67; 95% CI 0.49–4.85) were associated with improved MAR. The mothers with a formal education increased the MAR by 2.48 (95% CI 0.78–4.19) compared to those who did not have a formal education. The score of the household wealth index rose by 2.31 (coefficient 2.31; 95% CI 1.61–3.01) in MAR. The score of household food insecurity declined by 0.64 (−0.64; 95% CI −0.83–−0.29) in MAR. Binning indicated that the number of children aged 5 decreased by 5.48 (95% CI −7.74–−3.23) in the MAR for nutrients. The children’s age (*β*: 0.14; 95% CI 0.01–0.27) was positively significantly associated with MAR. However, variables such as the child’s sex, mother’s age, father's education, and family size did not have a statistical association with MAR (Table 4).

**Table 4:** Simple and multivariable linear regression analysis for factors associated with MAR of complementary food among children aged 6–23 months in Kombolcha, Northeast Ethiopia, 2023 (*n* = 255).

Variables and coding schemes	Category	Coefficients		
		Simple (bivariable analysis)	Multivariable (adjusted analysis)	
		*β* (95% CI)	*β* (95% CI)	*p*-value
Sex of children	Female	Reference		
	Male	16.99 (13.38, 20.61)	1.05 (−0.22, 2.33)	0.104
Age of children (6–23 months)		1.33 (0.92, 1.74)	0.14 (0.01, 0.27)*	0.027
Age of mothers (15–49 years)		1.01 (0.68, 1.34)	0.08 (−0.01, 0.17)	0.107
Mother's education status	0 = Informal	Reference		
	1 = Formal	19.30 (15.00, 23.61)	2.48 (0.78, 4.19)*	0.004
Father's education status	0 = Informal			
	1 = Formal	19.87 (13.58, 26.15)	0.29 (−1.87, 2.46)	0.790
Family size		−6.52 (−8.38, −4.66)	−0.20 (−0.87, 0.47)	0.558
Number of under-five children		−27.75 (−31.06, −24.42)	−5.48 (−7.74, −3.23)*	≤0.0001
Household wealth index		9.69 (7.99, 11.39)	2.31 (1.61, 3.01)*	≤0.0001
Household food insecurity access scale score (HFIAS)		−2.31 (−2.45, −2.16)	−0.64 (−0.83, −0.45)*	≤0.0001
Received information about the importance of dietary diversity during ANC follow-up	0 = No			
	1 = Yes	26.29 (22.68, 29.91)	3.26 (1.23, 5.29)*	0.002
Received information about the importance of dietary diversity during PNC follow-up	0 = No	Reference		
	1 = Yes	26.87 (22.89, 30.85)	2.67 (0.49, 4.85)*	0.016
Minimum meal frequency	0 = No	Reference		
	1 = Yes	11.75 (7.84, 15.66)	3.49 (2.12, 4.85)*	≤0.0001
Minimum dietary diversity	0 = No			
	1 = Yes	27.54 (25.06, 30.02)	9.74 (7.66, 11.82)*	≤0.0001
Minimum acceptable diet	0 = No			
	1 = Yes	27.32 (23.22, 31.41)	6.16 (4.12, 8.20)*	≤0.0001

*β* (95% CI) = coefficient with 95% confidence interval.

* *p* < 0.001 is statistically significant. ANC, antenatal care; PNC, postnatal care and variables included in the analysis: child age (in months), sex of the child, maternal education status, and household wealth index were considered natural confounder.

## Discussion

This study showed that all the complementary foods did not contain adequate amounts of protein, fat, energy, carbohydrate, folate, vitamin C, calcium, vitamin A, thiamin, riboflavin, niacin, vitamin B6, vitamin B12, iron, and zinc (values <1 or 100%) for children aged 6–23 months. This finding aligns with studies conducted in Jimma, Ethiopia, which reported that the diets of predominantly fed children lacked adequate protein, fat, carbohydrate, energy, and calcium as recommended for complementary feeding. However, iron and zinc levels were found to be adequate (1).

The computed adequacy ratio was almost consistent with similar studies conducted in Uganda, Ghana, and Zambia (22–24). However, the ratio was lower than that in a study conducted in the UK (25), which revealed that the majority of children have an adequate or more than adequate intake of energy, protein, and micronutrients. This may be due to the fact that the complementary foods are primarily grain-based. These grain-based foods contain high levels of phytic acid, which can inhibit the absorption of essential micronutrients such as iron, zinc, and calcium (9). Even though the introduction of animal-source foods, such as meat, fish, poultry, and eggs, into the diet is late, very few children consume them (1). This indicates that meeting the recommended intake for dietary adequacy has remained a challenge throughout the country. This study identified that the majority of the children consumed cereals and grains (91.4%) and legumes (70.2%), while the consumption of eggs (37.6%), dairy products (milk, yogurt) (43.1%), ﬂesh foods (meat, fish, and poultry) (26.3%), vitamin-A rich fruits and vegetables (32.2%), and other fruits and vegetables (35.5%) was low. This finding was almost consistent with studies form Southwest Ethiopia (1) and Bench Maji Zone, Southwest Ethiopia (26). However, this finding was slightly higher than the findings of a report from Southern Ethiopia (10). This may be because of differences in the season, study setting, and sample size of this study. The results of this study showed that the MAR was 63% (95% CI 60.8–65.14) among children aged 6–23 months in Northeast Ethiopia. The MAR in this setting was higher than findings from India (50%) (27), Bangladesh (43%) (28), and Madagascar (50%) (29). In contrast to the above findings, the magnitude of the MAR in our study was lower than reports from Malaysia (67%) (14). This may be due to the low nutrient density of their diet, coupled with consumption of small quantities of food with limited diversity. In addition, thin gruel made from a mixture of cereal and legumes was commonly consumed among children in Northeast Ethiopia, unlike children in some sub-Saharan Africa who were fed with cereal-based porridge (13, 29). The low MAR in the present study is an indication that children in the study area were not likely to meet the adequate macro- and micronutrient requirements for growth.

This study illustrated that dietary diversity was positively associated with nutrient adequacy.

This result was supported by studies conducted in Mali (29) and studies from developing countries (30, 31). Many dietary guidelines also recommend the inclusion of a variety of foods in the diet, which is associated with adequate intake of all the essential nutrients (4, 20).

Dietary intake adequacy in terms of MAR was shown to be more inﬂuenced by the minimum dietary diversity indicator. This ﬁnding was in line with a study carried out in Malaysia where the minimum acceptable diet in predicting dietary quality (14). The children need to be fed a wider variety of foods since an increase in individual minimum dietary diversity shows a corresponding increase in nutrient intake (1).

In this study, the minimum acceptable diet was statistically significant with a MAR. Similarly, the finding was supported by findings from EDHS 2016 where minimum acceptable diets were associated with better adequacy of diets for children (14, 32). A study conducted in Wolayita also showed a significant association between minimum acceptable diet and nutritional adequacy in children's diets (33). The possible justification for this may be related to the availability of diversified foods for children that satisfy essential nutrients required for growth and development, leading to improved nutritional adequacy.

This study found that meal frequency showed a positive association with the MAR of children. To meet the macro- and micronutrient requirements for the growth and development of infants and young children, WHO/UNICEF recommends a minimum meal frequency for children aged 6–23 months (34). This finding was consistent with findings from a study carried out in Uganda (22). The recommended number of meals per day for a healthy breastfed baby to meet nutritional adequacy should be 2–3 times at 6–8 months of age, 3–4 times at 9–11 months of age, and 3–4 times with 1–2 additional nutritious snacks at 12–23 months of age. This may be because as the meal frequency increases, the nutritional adequacy would be met, leading to an increase in the MAR.

The study observed that the household wealth index was a significant predictor of nutrient intake adequacy in terms of MAR. This finding is in line with studies conducted in Wolayita, Southern Ethiopia (33) and Nigeria (35). This is possibly because these children’s families may have afforded to purchase meat or to slaughter animals for their children. The rising income of the family increases the consumption of meat-based food, leading to nutrient adequacy being met.

In this study, food insecurity was negatively associated with the MAR. The studies conducted abroad showed the MAR as an index of household food insecurity (26, 31). Furthermore, local studies carried out in Ethiopia showed that food security was significantly associated with adequate diet quality (36). Other reasons for food insecurity may include low food supply, insufficient quality, lack of resources, and limited variety of foods due to a lack of resource because there was not enough food thereby decreasing nutrient quality.

Our study reported that having a formal education was positively associated with nutrient adequacy and is supported by other studies (37–39). This may be because education has a strong positive influence on adequate nutrient intake (8). Maternal education with a positive deviance from the norm may effectively inﬂuence the behavior of their partners. There is a reasonable body of evidence about the link between maternal knowledge of infant and young child feeding (IYCF) and the quality of the diet offered to children.

Our study found that information about the importance of dietary diversity during ANC and PNC checkups was positively associated with the adequacy of complementary food. Consistent ﬁndings have been reported by studies conducted in Ethiopia (1, 10, 13, 39), and Nepal (40). This may be because promoting the utilization of maternity services and stronger integration with IYCF helps to improve infant feeding practices. In addition, mothers who attended ANC and PNC visits may have better access to services or might be from a wealthier family and thus are more likely to provide a diet quality to their children (40).

The other factor that turned out to be a significant predictor of MAR was the number of children. Multiple studies from Ethiopia show that the number of children was a significant determinant of complementary food adequacy (1, 10, 13, 39). This could be because caregivers assume that younger infants do not need diverse foods or their gut may not be able to digest animal-source foods. Consequently, complementary feeding might be initiated with monotonous staples.

The study's practical and policy implications emphasize the need for enhanced nutritional education for mothers and caregivers, supported by health workers during ANC and PNC visits, to promote dietary diversity in complementary feeding. It calls for initiatives to improve food availability through local food production and community-based programs, alongside policy support for food security, including subsidies for nutrient-dense foods.

Using community-based quantitative methods is a strength of this study as it increases the validity of the data and helps to assess the nutrient adequacy of complementary foods in children aged 6–23 months. The use of a validated multiple-pass 24-h recall and data collection tool was pre-tested before the actual data collection time. We use salted replicas of local common infant and young child food to estimate portion size.

The present study has some limitations. First, the reported nutritional adequacy was assessed based on single-day recall; hence, it may not exactly reﬂect the common dietary practices of the children and it is possible that the dietary intake of the child may be affected by the appetite and health status of the child. However, such variables were not accounted for in the study. This may result in an underestimation of the association.

## Conclusion

The study concludes that the mean nutrient adequacy ratio of complementary foods among children aged 6–23 months in Northeast Ethiopia is alarmingly low, indicating a significant gap in meeting the nutritional recommendations set by the WHO. Various factors, including the child's age, maternal education, household wealth index, food insecurity, and dietary diversity, were identified as critical determinants of nutrient adequacy. These findings underscore the urgent need for comprehensive interventions at multiple levels, including education, food security, and health services, to enhance the consumption of adequate and diverse complementary foods. By addressing these factors, stakeholders can improve the nutritional status of young children, ultimately contributing to better health outcomes and development in the region.

## Data Availability

The datasets presented in this study can be found in online repositories. The names of the repository/repositories and accession number(s) can be found in the article/Supplementary Material.
