# Supplementary material for: Mean nutrient adequacy ratio and associated factors of complementary foods among children aged 6–23 months in Northeast Ethiopia
Source: Front Pediatr. 2025 Mar 7;13:1446431. doi: 10.3389/fped.2025.1446431 (PMC11925895; doi:10.3389/fped.2025.1446431)
Supplement: Supplementary file 1 [file Table1.docx]

**Supplementary file II: Result of equipment calibration**

| equipment | food type | weight (g)/unit of equipment |
| --- | --- | --- |
| spoon | flour used to make gruel | 41 |
|  | indamines raw | 14 |
|  | melted butter | 2 |
|  | stew made from legumes | 25 |
|  | sugar | 15 |
|  | Oil | 5 |
|  | papaya | 29 |
|  | salt | 21 |
|  | avocado | 19 |
|  | boiled rice | 24 |
| ladle | flour used to make gruel | 123 |
|  | stew made from legumes | 25 |
|  | stew made from potato | 100 |
|  | flour of whole maize | 125 |
|  | boiled rice | 150 |
| cup 1 (tasa) | barley | 650 |
|  | red sorghum | 775 |
|  | maize | 825 |
|  | red teff | 1000 |
|  | wheat | 950 |
|  | oats | 975 |
|  | round pea | 825 |
| Cup2 | tea | 125 |
|  | coffee | 115 |
|  | Oil | 105 |
|  | mango juice | 125 |
|  | milk | 110 |
| Cup3 | gruel | 250 |
|  | milk | 225 |
