# Supplementary material for: Mean nutrient adequacy ratio and associated factors of complementary foods among children aged 6–23 months in Northeast Ethiopia
Source: Front Pediatr. 2025 Mar 7;13:1446431. doi: 10.3389/fped.2025.1446431 (PMC11925895; doi:10.3389/fped.2025.1446431)
Supplement: Supplementary file 2 [file Table2.docx]

**Supplementary file III: result of market surveillance**

| food item | cost (birr) | weight (g)/package of food |
| --- | --- | --- |
| white bread | 3 | 250 |
|  | 5 | 300 |
| toasted white bread | 3 | 250 |
|  | 5 | 300 |
| ALPHA | 3 | 100 |
| Cappuccino biscuit | 3 | 100 |
| Dagim biscuit | 3 | 100 |
| Abu Woaladbiscuit 2 45 | 15 | 125 |
| glucose biscuit | 2 | 100 |
| indamine | 10 | 125 |
| Avocado | 2.5 | 45 |
| Egg | 4 | 38 |
| RANI | 20 | 200 |
| YAMI | 15 | 250 |
| Tropix Mango/Pirgate Juice | 20 | 296 |
