# Supplementary material for: Mean nutrient adequacy ratio and associated factors of complementary foods among children aged 6–23 months in Northeast Ethiopia
Source: Front Pediatr. 2025 Mar 7;13:1446431. doi: 10.3389/fped.2025.1446431 (PMC11925895; doi:10.3389/fped.2025.1446431)
Supplement: Supplementary file 3 [file Table3.docx]

**Supplementary file VI**: Mean micronutrient intake for age category in Northeast Ethiopia, 2023

| Nutrient | Age category | RDA | Mean intake |
| --- | --- | --- | --- |
| Calorie(kcal/d) | 6-8 | 269 | 210.7±10.01 |
|  | 9-11 | 451 | 383.3±11.6 |
|  | 12-23 | 746 | 717.7±6.2 |
| Fat | 6-8 | 28 | 22.8.±1.0 |
|  | 9-11 | 29 | 26.2±0.74 |
|  | 12-23 | 33 | 32.5±0.4 |
| Protein(g/d) | 6-8 | 13.7 | 10.6±0.55 |
|  | 9-11 | 14.9 | 12.4±0.38 |
|  | 12-23 | 14.5 | 13.6±0.28 |
| Carbohydrate | 6-8 | 60 | 36.3±1.7 |
|  | 9-11 | 95 | 61.9±1.69 |
|  | 12-23 | 130 | 98.7±1.4 |
| Vitamin A-Pre (RE/d) | 6-8 | 350 | 156.9±9.2 |
|  | 9-11 | 350 | 178±6.1 |
|  | 12-23 | 400 | 224.2±4.66 |
| Vitamin B1(mg/d) | 6-8 | 0.3 | 0.13±0.01 |
|  | 9-11 | 0.3 | 0.14±.011 |
|  | 12-23 | 0.5 | 0.26±.009 |
| Vitamin B2(mg/d) | 6-8 | 0.5 | 0.21±0.01 |
|  | 9-11 | 0.5 | 0.22±.015 |
|  | 12-23 | 0.8 | 0.44±.008 |
| Vitamin B3(mg/d) | 6-8 | 5.4 | 2.3±0.14 |
|  | 9-11 | 5.4 | 2.5±.10 |
|  | 12-23 | 9 | 5.2±0.087 |
| Vitamin B6(mg/d) | 6-8 | 0.3 | 0.10±0.013 |
|  | 9-11 | 0.4 | .19±.014 |
|  | 12-23 | 0.7 | .42±.01 |
| Vitamin B12(µg/d) | 6-8 | 0.1 | 0.04±0.005 |
|  | 9-11 | 0.1 | .05±.004 |
|  | 12-23 | 0.5 | 0.29±.008 |
| Vitamin C (mg/d) | 6-8 | 20 | 9.9±0.38 |
|  | 9-11 | 20 | 11.2±0.22 |
|  | 12-23 | 20 | 13.1±0.18 |
| Float(µg/d) | 6-8 | 32 | 17.3±1.3 |
|  | 9-11 | 32 | 19±1.14 |
|  | 12-23 | 50 | 35.2±0.8 |
| Calcium(mg/d) | 6-8 | 600 | 279.1±15 |
|  | 9-11 | 600 | 298.8±11.8 |
|  | 12-23 | 400 | 260.4±7.5 |
| Iron(mg/d) | 6-8 | 8.5a | 3.5±0.2 |
|  | 9-11 | 8.5a | 3.7±.14 |
|  | 12-23 | 5a | 3.2±.09 |
| Zinc(mg/d) | 6-8 | 5.6b | 2.4±0.12 |
|  | 9-11 | 5.6b | 2.4±.10 |
|  | 12-23 | 5.5b | 3.2±.07 |

WHO/UNU, 2003)(20). a: Iron RNI Based on a 15% and b: Zinc RNI Based on a moderate bioavailability level

**Key**: RDA, Recommended allowance
